# Combined Species Identification, Genotyping, and Drug Resistance Detection of *Mycobacterium tuberculosis* Cultures by MLPA on a Bead-Based Array

**DOI:** 10.1371/journal.pone.0043240

**Published:** 2012-08-20

**Authors:** Indra Bergval, Sarah Sengstake, Nadia Brankova, Viktoria Levterova, Edgar Abadía, Nino Tadumaze, Nino Bablishvili, Maka Akhalaia, Kiki Tuin, Anja Schuitema, Stefan Panaiotov, Elizabeta Bachiyska, Todor Kantardjiev, Rina de Zwaan, Anita Schürch, Dick van Soolingen, Anja van ‘t Hoog, Frank Cobelens, Rusudan Aspindzelashvili, Christophe Sola, Paul Klatser, Richard Anthony

**Affiliations:** 1 KIT Biomedical Research, Royal Tropical Institute, Amsterdam, The Netherlands; 2 National Center of Infectious and Parasitic Diseases, Sofia, Bulgaria; 3 Institute of Genetics and Microbiology UMR 8621 CNRS/UPS11, Orsay, France; 4 Venezuelan Institute of Scientific Research, Caracas, Venezuela; 5 National Tuberculosis Reference Laboratory, National Center for Tuberculosis and Lung Diseases, Tbilisi, Georgia; 6 MRC-Holland, Amsterdam, The Netherlands; 7 Tuberculosis Reference Laboratory, Centre for Infectious Disease Control, National Institute for Public Health and the Environment, Bilthoven, The Netherlands; 8 Departments of Microbiology and of Pulmonary Diseases, Radboud University Nijmegen Medical Centre/University Lung Centre Dekkerswald, Nijmegen, The Netherlands; 9 Amsterdam Institute of Global Health and Development, Amsterdam, The Netherlands; St. Petersburg Pasteur Institute, Russian Federation

## Abstract

The population structure of *Mycobacterium tuberculosis* is typically clonal therefore genotypic lineages can be unequivocally identified by characteristic markers such as mutations or genomic deletions. In addition, drug resistance is mainly mediated by mutations. These issues make multiplexed detection of selected mutations potentially a very powerful tool to characterise *Mycobacterium tuberculosis*. We used Multiplex Ligation-dependent Probe Amplification (MLPA) to screen for dispersed mutations, which can be successfully applied to *Mycobacterium tuberculosis* as was previously shown. Here we selected 47 discriminative and informative markers and designed MLPA probes accordingly to allow analysis with a liquid bead array and robust reader (Luminex MAGPIX technology). To validate the bead-based MLPA, we screened a panel of 88 selected strains, previously characterised by other methods with the developed multiplex assay using automated positive and negative calling. In total 3059 characteristics were screened and 3034 (99.2%) were consistent with previous molecular characterizations, of which 2056 (67.2%) were directly supported by other molecular methods, and 978 (32.0%) were consistent with but not directly supported by previous molecular characterizations. Results directly conflicting or inconsistent with previous methods, were obtained for 25 (0.8%) of the characteristics tested. Here we report the validation of the bead-based MLPA and demonstrate its potential to simultaneously identify a range of drug resistance markers, discriminate the species within the *Mycobacterium tuberculosis* complex, determine the genetic lineage and detect and identify the clinically most relevant non-tuberculous mycobacterial species. The detection of multiple genetic markers in clinically derived *Mycobacterium tuberculosis* strains with a multiplex assay could reduce the number of TB-dedicated screening methods needed for full characterization. Additionally, as a proportion of the markers screened are specific to certain *Mycobacterium tuberculosis* lineages each profile can be checked for internal consistency. Strain characterization can allow selection of appropriate treatment and thereby improve treatment outcome and patient management.

## Introduction

Effective treatment of patients infected with (drug-resistant) TB relies on accurate diagnosis and appropriate therapy. It is therefore crucial to confirm and characterise the species present in sputum cultures [1] as well as to detect drug resistance at an early stage. Unfortunately in many high burden settings culture of sputum samples, if performed at all, is not followed by further molecular characterisation [2]. This can lead to suboptimal treatment and patient management.

Over the last years a diverse range of molecular tools have been developed to characterise and type *Mycobacterium tuberculosis* complex. Only a proportion of these methods, based on identification/detection of CRISPRs (spoligotyping), insertion sequences (IS6110 Restriction Fragment Length Polymorphism (RFLP)), large sequence polymorphisms (LSPs), Regions of Difference (RD) typing or tandem repeats (Mycobacterial Interspersed Repetitive Units-Variable Number of Tandem Repeats (MIRU-VNTR) have been widely applied [3]–[7].

Genetic information derived by these typing methods from tuberculosis (TB) strains all over the world has revealed the clonal architecture, the phylogeography and evolutionary descent of different strains [7]–[13]. The challenge with monomorphic bacteria is that they contain so little sequence diversity that sequencing a few gene fragments yields little or no information thereby making it difficult to identify variable regions suitable for epidemiological studies. However, for genotyping purposes this clonal population structure is quite advantageous, as transfer of DNA does not occur and the accumulated genetic changes are fixed, which can be used to unequivocally identify specific lineages [9], [14]. Drug resistance mutations are observed in multiple lineages because they arose under strong selective pressure whereas mutations occurring through random genetic drift are fixed and unique to specific lineages [9].

Drug resistance in *M. tuberculosis* isolates is largely due to a limited diversity of mutations [15]–[19] and not the acquisition of (plasmid-mediated) resistance genes. Detection of clustered drug resistance mutations in *M. tuberculosis* by sequencing [20], reverse hybridisation to low-density arrays [21], [22] or molecular beacons in a PCR reaction [23], [24] has been extraordinarily successful. These methods form the theoretical basis for almost all currently used molecular methods for the detection of drug resistance in *M. tuberculosis*. Unfortunately, although very effective, the methods used to date do not naturally lend themselves to highly multiplexed detection of dispersed markers and full genetic characterization of *M. tuberculosis* isolates therefore requires multiple tests.

Traditionally the diagnosis, culture and typing of *M. tuberculosis* relies on dedicated methods, requiring specially trained workers and specially equipped laboratories. This situation persists because of the slow growth and need for specialised media and laboratory safety infrastructure. In areas where TB is less prevalent or where TB is prevalent but resources are too limited to have dedicated staff and laboratories, this can be a problem. One consequence is that molecular typing and, to some extent, determination of drug resistance are often performed retrospectively and generally only routinely performed in supra-national centers.

In the last decade there has been an explosion of genome sequences from large numbers of *M. tuberculosis* strains, revealing the presence of many lineage-specific genetic markers, such as unique SNPs and regions of difference (RDs), in addition to the markers which are currently in use [6], [8], [10]–[13], [25], [26].

As outlined above, the population structure of *M. tuberculosis* is clonal with no evidence of horizontal gene transfer. Markers (SNPs or deletions) associated with resistance or specific for distinct lineages can therefore be unequivocally identified and defined, making multiplexed detection methods for characterization of *M. tuberculosis* isolates very appealing.

Methods for multiplex SNP detection are available but have only rarely been developed for typing bacterial pathogens [27]–[30]. Here we report a novel multiplexing assay, allowing the simultaneous detection of an extensive panel of both drug resistance and genotypic markers in *M. tuberculosis* isolates. We used an established method of multiplex SNP typing which we have previously shown to be suitable for use with *M. tuberculosis*, the MLPA [28], [31]. This method was adapted so results can be analysed using a recently released liquid array reader based on a flow cell and CCD imaging (MAGPIX, Luminex, Austin, USA). We believe this technology offers a robust and potentially cost effective platform appropriate for use in tuberculosis laboratories performing culture.

The MAGPIX platform allows up to 50 analytes to be tested in a single tube per sample. We thus designed MLPA probes to target 47 informative markers and three internal controls. Included markers were selected based on the previous assay [28], published literature, or *in silico* searches. Markers targeting drug resistance associated mutations, mycobacterial species specific regions, *M. tuberculosis* genotype specific markers, as well as markers identifying epidemic strains, were included in the assay to demonstrate the versatility of this approach for the characterization of *M. tuberculosis* isolates. The method was validated using a panel of 88 selected well-characterised strains derived from various regions of the world, consisting of *M. tuberculosis* complex strains and non-tuberculous mycobacteria.

## Methods

### MLPA assay

**Figure 1:**
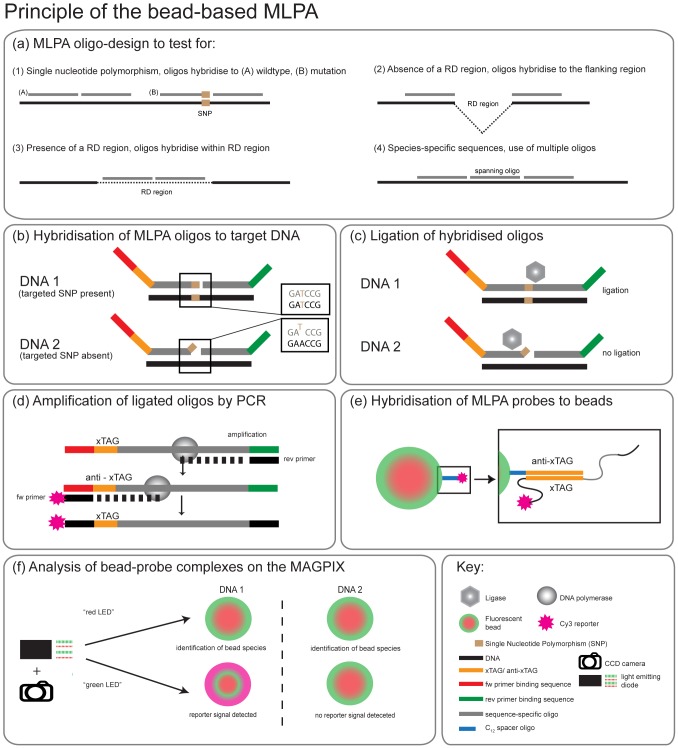
Overview of the bead-based Multiplex Ligation-dependent Probe Amplification (MLPA) assay. (a) MLPA oligo design. MLPA oligos were designed to test for (1) single nucleotide polymorphism, the absence (2) or presence (3) of a region of difference (RD), (4) species-specific sequences (b) Hybridisation of MLPA oligos to target DNA. Sequence-specific sequences hybridise to target DNA (DNA1 and DNA2). Each probe consists of a target-specific sequence (grey bars), a unique xTAG (orange bar), forward and reverse primer binding sequences (red and green bars). The MLPA oligos perfectly match to the sequence of DNA1 that harbours a SNP but not to DNA2. (c) Ligation of hybridised oligos. Only oligos that are hybridised directly adjacent to each other are ligated. (d) Amplification of ligated oligos by PCR. All ligated oligos are amplified in a PCR reaction using a single Cy3-labelled forward primer and unlabelled reverse primer. (e) Hybridisation of MLPA products to beads. Amplified probes hybridise to their anti-xTAG coupled to an individual bead species. (f) Analysis of bead-probe complexes on the MAGPIX. A red light emitting diode (LED) and a CCD camera identify first the individual bead species before green LEDs excite the reporter molecules on the probes. The signal is translated into Median Fluorescence Intensity (MFI). For DNA1 a reporter signal is detected on the bead species indicating the presence of the SNP, thus a mutation, in the respective DNA.

A schematic overview of the MLPA assay is shown in Figure 1.

In this study, the analysis of amplified MLPA probes was performed using the Luminex xTAG technology on the MAGPIX platform, a compact and robust device. Read-out is facilitated by a unique xTAG (24 nt) present on each amplified MLPA probe, which is complementary to the anti-xTAG on one bead in the array. The MAGPIX unlike previous Luminex readers is based on a CCD camera and LED illuminated flow cell rather than a flow cytometer. All MLPA probes and reagents were manufactured and supplied by MRC-Holland (Amsterdam, The Netherlands).

#### MLPA reaction

MLPA was performed according to the One-Tube MLPA Protocol for DNA detection and Quantification developed by MRC-Holland (www.mlpa.com) and as previously described [28] except that in this study MLPA probes were denatured for 10 minutes at 98°C before the overnight hybridisation step and MLPA probes were amplified by PCR (35 cycles of 30s at 95°C, 30s at 60°C and 45s at 72°C). MLPA products were analysed using the bead-based Luminex xTAG/MAGPIX system. MLPA probes were used in a concentration of 2 fmol of each probe per MLPA reaction. For amplification of ligated probes 2 µl of primermix (3.2 µM or 6.4 µM Cy3-labelled forward primer, 1.6 µM reverse primer, 4 mM dNTPs) was used per reaction. All steps of the MLPA were performed in a single tube for each sample using a single thermocycler program. Three extra samples were included in every experiment to monitor the quality of the assay: 1) a negative control (no DNA template or MLPA probes) to detect contamination with amplified MLPA products, 2) a contamination control to detect contamination with DNA template containing all reagents and MLPA probes, and DNA from a species other than MTBC and 3) a positive assay control containing all reagents, MLPA probes and template DNA from a previously characterised laboratory strain RB14 ([28] and Table S1).

From all DNA samples, irrespective of the DNA extraction procedure or concentration, 3 µl of template DNA was used for the MLPA assay. Agarose gel electrophoresis was used for confirmation of successful amplification but not for marker discrimination since all amplicons are approximately the same length.

#### Analysis of the MLPA products

The Luminex xTAG technology combined with the MAGPIX platform allows multiplexed analysis of up to 50 targets in a single assay. In this study we aimed to use the full multiplexing capacity of the MAGPIX and designed probes for the analysis of 47 mycobacterial targets and three controls.

The three controls included were: 1. The LumQ control is used to monitor the quantity of template DNA. It consists of two random oligos of 74 and 80 nt, both of which contain forward and reverse primer sites and the same xTAG. The LumQ oligos do not require ligation, they are ready to be amplified by PCR. However they are present in a very low concentration compared to the concentration of each MLPA oligo in a sample. If the MFI signal from the LumQ bead is low (MFI signals ≤50), this indicates that enough (target) DNA was present and the ligation reaction was successful. If the LumQ signal is high (MFI signals ≥300), this indicates that not enough (target) DNA was present or that the ligation reaction failed. 2. The LumD control indicates efficient denaturation and ligation (Table 1). Like the other MLPA probes it consists of two oligos each with a primer site and one with a unique xTAG. One of the LumD oligos targets a GC-rich sequence of the *recA* gene present in all members of the MTBC. This oligo has the highest GC content of all probes in the assay (GC-content of 91%). If the mycobacterial DNA denaturation was successful, a signal will be obtained from the LumD-specific beads. Both the LumD and LumQ controls are added to the samples along with the MLPA probes at a concentration of 2.4 fmoles/sample and 36 zeptomoles/sample, respectively. 3. The LumH control indicates adequate hybridisation of the MLPA products to the beads. The LumH control does not require ligation or amplification and consists of a single short Cy3-labelled oligo with an xTAG sequence binding to a unique bead species (Table 1). An amount of 1 pmoles of LumH control is added to each sample with the bead mix directly prior to analysis on the MAGPIX device.

**Table 1:** Summary of the MLPA probes designed and used in this study^a^.

Probes	Target	Target-specific sequence (5′–3′)	Target or information provided [ref]
embB-306	wt	[28]	EMB resistance marker
katG-315	mut (AGC 315 ACC)	[28]	INH resistance marker
inhA-15	mut (**−15** C/T)	[28]	INH resistance marker
rpoB-176	mut (GTC 176 TTC)	[28]	RIF resistance marker
rpoB-522	mut (TCG 522 TTG)	[28]	RIF resistance marker
rpoB-526G	mut (CAC 526 GAC)	[28]	RIF resistance marker
rpoB-526T	mut (CAC 526 TAC)	[28]	RIF resistance marker
rpoB-531	mut (TCG 531 TTG)	[28]	RIF resistance marker
gyrA-90	mut (GCG 90 GTG)	GCACCAGGCTGTCGTAGATCGACA	FLQ resistance marker [74]
		*CGTCGCCGTGCGGGTGGTAGTTGCCCATGGTCTCG*	
gyrA-94	mut (G**A**C 94 G**G**C)	CACGGCGACGCGTCGATCTACGG	FLQ resistance marker [53]
		*CAGCCTGGTGCGCATGGCCCAGCCCTGGT*	
rrs-1401	wt	CGGGCCTTGTACACACCGCCCGTCA	AMK/KAN/CAP resistance marker [51], [52]
		*CGTCATGAAAGTCGGTAACACCCGAAGCCAGTGGCCTAACCCTCGGGAGG*	
rrs-1402	mut (**1402 C/T**)	CTTCGGGTGTTACCGACTTTCATGACA	CAP resistance marker [51], [53]
		*TGACGGGCGGTGTGTACAAGGCCCGGGAACGTATTCACCGCAGCG*	
rpsl-43	wt	CCCGCGTGTACACCACCACTCCGAA	S resistance marker [75]
		*GAAGCCGAACTCGGCGCTTCGGAAGGTTGCCCGCG*	
MTBC 16S rRNA	MTBC-specifc sequence	[28]	16S rRNA gene, *M. tuberculosis* complex specific
mutT2-58	mut (GGA 58 CGA)	[28]	Genotype marker, specific for Beijing K1, V+, SA+ [33], [34]
ahpC-46	mut (**−46** A/G)	CGGCGATGCCGATAAATATGGTGTA	Genotype marker, specific for Central Asian [45]
		*ATATATCACCTTTGCCTGACAGCGACTTCACGGC*	
ogt-15	mut (AGC 15 ACC)	[28]	Genotype marker, specific for Haarlem
Ag85C-103	mut (GAG 103 GAA)	GACATCAACACCCCGGCCTTCGAA	Genotype marker, specific for LAM [65]
		*GAGTACTACCAGTCAGGGTTGTCGGTGATCATGC*	
RD9	targeting the absence of the RD	CCGCCGAAAATTACTACCGGAGCA	Genotype maker, RD present in animal strains and *M. africanum* West-African 1/2 [6], [26]
		*GCGCCCGTGTCGTCCACGGCTGCGATTATTGCCT*	
recC-1491	mut (**1491** C/G)	CCGTCGTGCACAACACGTGGCGGTTG	Genotype marker, specific for X family [66]
		*GGACTCGACCGCATCCTCACCGGGGTGGCCATGTC*	
fbpB-238	mut (CCC 238 CCA)	CCGGGTGTTGTTTGCGACCAGCTTT	Genotype marker, specific for Beijing K1, V+, V−, SA+ [33], [34], [76]
		*GGGATCTGCTGCGTAGGGTCGTTGCGCTCCCATGCCGG*	
RD702	targeting the absence of the RD	GAGCTCGACCCGCTCGAATCCCAGCAA	Genotype marker, RD present in *M. africanum* West-African 2 [77], [78]
		*CCGCAACCGCGATTCGCCGGGAGCTGCGATCATATACAGCTG*	
RD10	targeting the presence of the RD	TTACCCCGCCACCGACGTTCATGAA	Genotype marker, RD present in *M. microti, M. bovis, M. pinnipedii, M. africanum West-Africanum 2*
		*TCGGATAAAGAAGCCGTTGTCCAAGCCCTCGGGACGGCCGCCG*	[6], [78]
acs-1551	mut (**1551 G/**A)	CGCTCGCATAGACCCCGACGGCGCA	Genotype marker, specific for Beijing SA+ [33], [34]
		*ATCTGGGTACTAGGCCGCATCGACGACGTGATGAACGTGTCCGGG*	
RD131	targeting the absence of the RD	GAACGCCATGATTTCCTGGTCGGAA	Genotype marker, RD present in Beijing K1 [64]
		*AGCCGGTCGCCGTCGAGCTCGGCTGCCAGCAGTGC*	
RD1-BCG	targeting the presence of the RD	ACGTCGTGCTTCTGGTCGACGATTGGCACA	Genotype marker, RD present in *M. bovis* BCG [79]
		*TCCAGCCGCCCGGATCCAGCATCTGTCTGGCATAGCTGCC*	
TbD1	targeting the presence of the RD	[28]	Genotype marker, present in "ancestral" TB
alkA-260	mut (CTG 260 CTA)	CCTGCTTGTGCTCGATGATTTCCGCGACCTA	Genotype marker, specific for *M. bovis* [66]
		*ATGACGGCCACTGCACGTTGCCGACGGCTGCTG*	
RD12can	targeting the presence of the RD	CCGCTATGCCACCCGTTAGGGCTCGA	Genotype marker, RD present in *M. canetti* [6], [80]
		*CGCCCGTGCGCCATTCGTGGCGACAACTGC*	
RD1-mic	targeting the presence of the RD	CTGTTCAAACAAATCAGCGACAAAATGGGACTCGGC	Genotype marker, RD present in *M. microti* [79]
		*GCAGCGCGCGCTCAGCGACGCCGACTGGCAT*	
ESAT-6	targeting the presence of ESAT-6	CTGGGGCGGTAGCGGTTCGGAGGCG	Genotype marker, target sequence not present in *M. microti, M. bovis (BCG)* [79]
		*TACCAGGGTGTCCAGCAAAAATGGGACGCCACGGCTACCGAGCTG*	
RD2seal	targeting the presence of the RD	GAACATCCGCGAGCAGGCGATCGCCA	Genotype marker, RD present in *M. pinnipedii* [81]
		*CGAGATGCCGATCCGGCGCCCAAATGCGGC*	
RD105	targeting the presence of the RD	GGCGTGCCGTCGCGCCCGTCCCA	Genotype marker, RD present in all Beijing and East Asia "Non-Beijing blue"
		*CGGCGACCACGGCGTCCACCGGGTCCAC*	[25], [35]
RD239	targeting the presence of the RD	TGTAATAATGACCGACGGCCGCATGCTA	Genotype marker, RD present in East African Indian [25]
		*CCGTTGCTTGTCGTGAACGGTTTGACGGTGATCCG*	
pks15/1–7	targeting the presence of the 7bp RD	GGTTGAGGCGAGCGAAAGCACCGGG	Genotype marker, present in "modern" TB [81]
		*GGCCGCGGCCGTCGATGGTGCCGTG*	
RD750	targeting the presence of the RD	GTGGTTGCCTCGCATGCGACGGAGTGCA	Genotype marker, RD present in Central Asian [26]
		*TTGGTACTTGCATGTGCGGCATGCGGTGGGCG*	
pckA-1119	mut (**1119 C/**T)	GCATTGGTTTCCGTCTCGCGGAAGTACCAA	Genotype marker, specific for Beijing SA-, CHIN- [33], [34]
		*TCGTTGCCCTTCCAGTCGATCAGGTGCTGCGGGTCG*	
NTM 16S rRNA	NTM-specific sequence	GAATAGGACCACGCGCTTCATGiiiTGTGGTGGAAAGC	16S rRNA gene, specific for non-tuberculous mycobacteria
		*TTTTGCGGTGTGGGATGGGCCCGCGGCCTATCAGCTT*	
NTM^kans^	species-specific sequence	GCGAGCATCAAATGGATGCGTTGCCCTA	target specific for *M. kansasii* (GenBank ID: AB026695.1)
		*CGGGTAGCGTGTTCTTTTGTGCAATTTTATTCTTTGGTTTTTGTG*	
NTM^xen^	species-specific sequence	CACTGTTGGGTTTTGAGGCAACACCC**GTGGTGGTGTTGTGCTCCGCG**	target specific for *M. xenopi* (GenBank ID: Y14190.1)
		*TGGTGGCGGGGTGTGGTGTTTGAGTGTTGG*	
NTM^aviC^	species-specific sequence	CAGGGCCCAATAGTGTGTCTGGCAAT	target specific for *M. avium* complex (GenBank ID: AY859046.1)
		*CATTTGCTGTTGCGCACCCGGCCCCCGTCCACTACAGACGGGAACC*	
NTM^avi^	species-specific sequence	AAGGAGCACCACGAAAAGCACCCCAAC**TGGTGGGGTGCGAGCCGTGAGGGGTTC**	target specific for *M. avium* subsp. *avium* (GenBank ID: EF059906.1)
		*CGTCTGTAGTGGACGGGGGCCGGGTGCGCAAC*	
NTM^fort^	species-specific sequence	TTGGGGGGTGGCATCCGGTTGC**GGGTGTCGGCGTGTTGTTGCCTCA**	target specific for *M. fortuitum* (GenBank ID: AJ291588.1)
		*CTTTGGTGGTGGGGTGTGGTGTTTGATTTGTGGATAG*	
LumD	internal control	GACATCGTGGTGATCGACTCGGT	recA gene, *M. tuberculosis* specific
		*GGCGGCGCTGGTGCCGCGCGCGG*	
LumH	internal control	GATGAATATAGTAAGTATTGAGTA	random oligo

a only probes that were functional in this study are shown. Probes are named after the gene and specific codon, nucleotide position (bold), or region they target. Probes are either targeting the mutation (mut) or the wild type (wt) sequence or the presence or absence of an RD. Bacterial DNA sequences are targeted with the left oligo (capital letters), spanning oligo (bold), right oligo (italics), iii =  inosine. xTAG sequences are not shown. RD  =  region of difference.

For the analysis of the amplified MLPA products using the bead-based array, we used an adapted version of ‘Sample protocol for direct DNA hybridisation – Washed Assay Format using magnetic microspheres’ provided by Luminex. (http://www.luminexcorp.com/prod/groups/public/documents/lmnxcorp/washed-direct-dna-magnetic.pdf) last access 20-06-2012. We used Cy3-labelled MLPA primers, eliminating the need for an additional hybridisation step to the reporter dye streptavidin-phycoerythrin, thereby reducing the hands-on time. Briefly, 10 µl of MLPA product per sample was hybridised to 500 beads of each bead species in a total volume of 50 µl (33 µl 1.5 x TMAC buffer (5M TMAC, 20% Sarkosyl, 1M Tris-HCL pH 8.0, 0.5 M EDTA pH 8.0, beads resuspended therein), 7 µl of Tris-EDTA (1 mM EDTA, 10 mM Tris-HCl, pH 8.0) and 20 nM LumH control). MLPA products were denatured in a thermocycler for 10 min at 95°C followed by hybridisation to the beads for 30 min at 45°C. This mixture was directly analysed on the MAGPIX equipment without additional washing. A minimum of 35 beads per species were counted and analysed per sample. A beads-only control (extra sample), was included each time the assay was run for background signal analysis. Median fluorescence Intensity (MFI) signals from the samples were measured on the MAGPIX equipment using the xPonent 5.1 software (Luminex, Austin, TX, USA).

#### Data analysis

**Figure 2:**
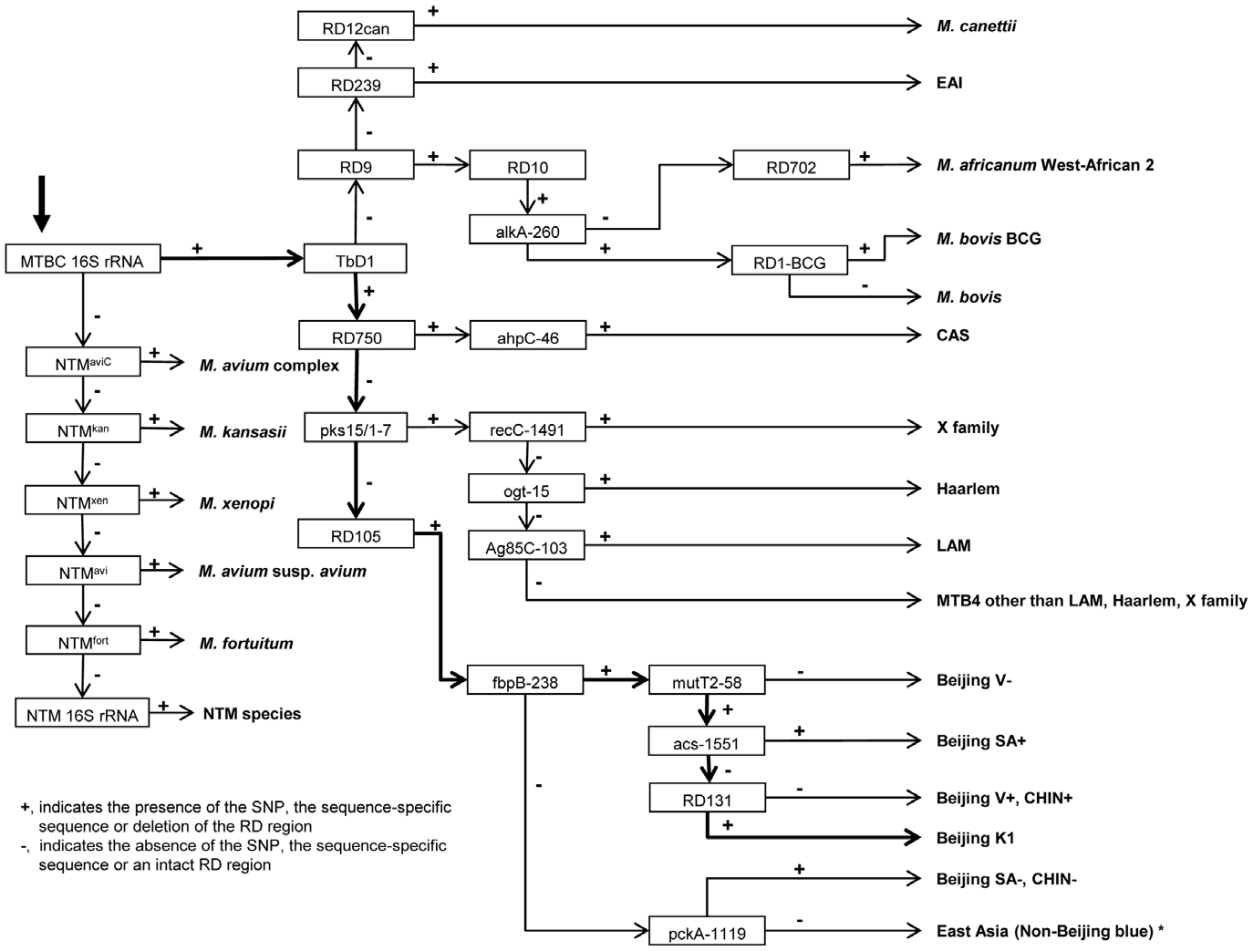
Algorithm applied to all strains analysed for species identification of *M. tuberculosis* complex and non-tuberculous mycobacteria. MLPA markers are framed and final NTM species, MTBC lineages or sublineages are shown in bold. The species identification of a sample always starts with the MTBC 16SrRNA marker. As an example the call for the Beijing lineage K1 is highlighted with bold arrows. The following markers are present or absent in a strain belonging to the Beijing K1 lineage: MTBC 16S rRNA (present), TbD1 (present), RD750 (absent), pks15/1–7 (absent), RD105 (present), fbpB-238 (present), muT2-58 (present), acs-1551 (absent), RD131 (present). * as defined in [25].

Based on preliminary testing of DNA from MTBC cultures a Median Fluorescence Intensity (MFI) value of 150 was selected as a threshold value for all markers. Automated calls were set up for the identification of MDR-TB, XDR-TB, *M. tuberculosis* lineages, sublineages and NTM on the basis of algorithms and comprehensive MTBC phylogeny (Figure 2). Data analysis was performed using a dedicated Excel spreadsheet (Microsoft Seattle, USA). The csv-file produced by the xPonent software was imported into the Excel worksheet. The profiles were first checked for validity; inclusion criteria were a MFI signal for MTBC 16S rRNA-specific beads equal to or higher than 150 or an MFI signal from an NTM bead equal to or higher than 150. Signals from other beads were then considered positive if the MFI measured was equal to or greater than 150 and conditional formatting was used to highlight this signal.

**Figure 3:**
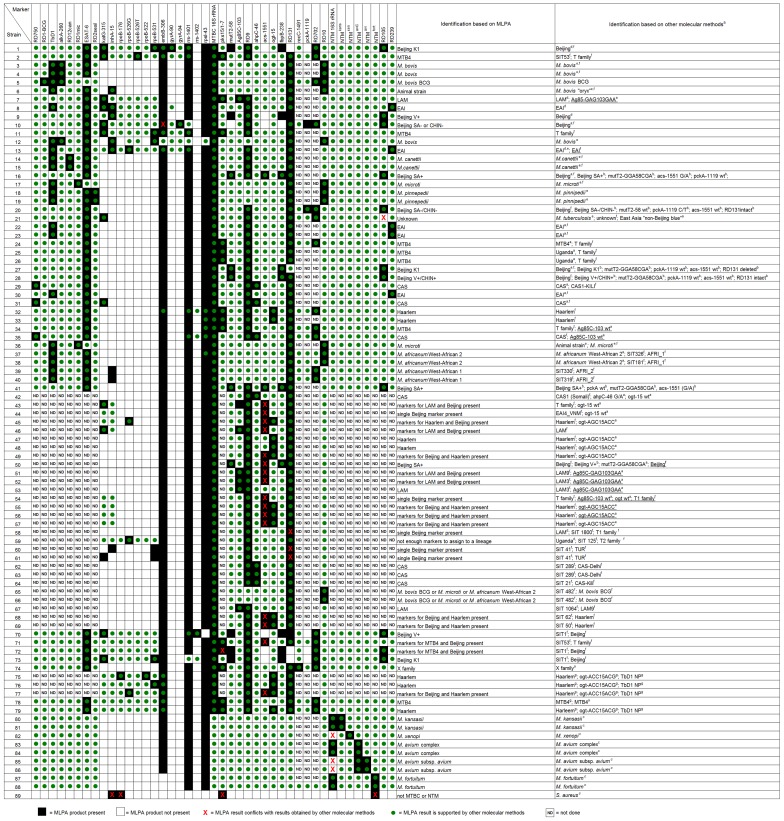
Validation of MLPA probes on 88 previously characterised mycobacterial strains. The MLPA was performed on 79 *M. tuberculosis* isolates (strains 1–79), nine non-tuberculosis mycobacteria (strains 80–88) and one species unrelated to mycobacteria (strain 89). Species identification was determined on the basis of the presence or absence of MLPA markers following calls mentioned in Figure 2. Results obtained by MLPA were compared to results obtained from testing the same strain by other molecular methods. ^a^Strain-specific drug resistance profiles and genotypic information obtained by other molecular methods is available in Table S1. The presence or absence of an MLPA product is indicated with a black square or a white square, respectively. The confirmation of the MLPA result by other molecular methods is indicated with a green dot; conflicting results between MLPA and other molecular methods are indicated with a red cross. ND =  Analysis for this marker was not done. MTB4 is defined as *M. tuberculosis* group 4 [26] but not X family, LAM or Haarlem.

We thus obtained an MLPA profile from every analysed strain which is reported in Figure 3 (black squares indicate the presence of an MLPA product, white squares indicate the absence of an MLPA product). The correlation of this profile with the previously collected data was also assessed. MLPA results (positive or negative) that were supported by previously collected data (Table S1) are indicated with a green dot in Figure 3 along with any results that directly contradict or conflict with previously collected data (Figure 3, indicated with a red X). MLPA results that do not conflict with previously available data but are not directly confirmed by this data are regarded as unsupported and merely reported as a black or white square in Figure 3.

Although T strains may be members of multiple sublineages [32], they are always expected to be members of the MTB4 lineage. Therefore the assignment of a strain to MTB4, as classified by MLPA by presence of pks-15/1, is considered to be supported if previously determined T family by spoligotyping.

Any strain assigned to one of the Beijing lineages by MLPA could only be confirmed for strains where sequencing data was available; only sequencing has the ability to assign a strain to the Beijing lineages K1, V−, V+, SA−, SA+, CHIN−, CHIN+ [33], [34]. In this study, a strain is identified as Beijing if the RD105 region is deleted and at least one other Beijing marker is present [35]. If a strain was identified as Beijing by both MLPA and spoligotyping we considered the Beijing marker RD105 confirmed. If genotypic information was available from two methods, sequencing was chosen over spoligotyping and spoligotyping over MIRU-VNTR to be compared to the MLPA results obtained.

### Selection of strains/DNA targets

The MLPA assay was evaluated using 79 selected *M. tuberculosis* complex strains, nine non-tuberculous mycobacterial (NTM) strains and one other unrelated bacterial species (Table S1). This panel of tested strains consisted of cultured clinical isolates and previously described laboratory-generated mutants [28]; they were selected so that the wildtype and the mutant form of all targeted loci (Table 1) were represented at least once. Strains 1–4, 6–57, 62–69, 74, 80–87 were provided by the Tuberculosis Reference Laboratory of the National Institute for Public Health and the Environment, Bilthoven, The Netherlands. Strains 58–61 were provided by the National Center of Infectious and Parasitic Diseases, Sofia, Bulgaria. All Bulgarian strains were identified as MDR-TB on the basis of drug susceptibility testing (BACTEC MGIT 960, BD Sparks, MD, USA) and/or reverse hybridisation assays (MTBDR*plus* and MTBDR*sl*, Hain Lifescience GmbH, DE). Strains 70–73 were provided by the National Reference Center for Tuberculosis, Tbilisi, Georgia. Strains 5, 75–79, 88–89 were from KIT Biomedical Research, Royal Tropical Institute, Amsterdam, The Netherlands. A summary of the selected strains and strain-specific information available is shown in Table S1.

#### DNA isolation

DNA from bacterial cultures grown from single colonies was obtained by thermolysis [28], cetyltrimethylammonium bromide (CTAB) procedure [36] or column-based purification (QIAGEN Benelux B.V., Venlo, The Netherlands).

#### Sequencing of targeted loci

In some cases insufficient background information on the strain was available and DNA sequences targeted by MLPA probes were sequenced in selected strains from the collection to resolve contradictory results (Table S1 and Figure 3). The following primers were used to amplify and sequence selected regions targeted by the MLPA probes: gyrA-Forward (FW), 5′- ACTATGCGATGAGCGTGATCG-3′ and gyrA-Reverse (RV), 5′-ATGAAATCGACTGTCTCCTCGTCG-3′ (*gyrA* codon 90, 94); rrs-FW, 5′-ATCGCAGATCAGCAACGCTGC-3′ and rrs-RV, 5′- ACGGCTACCTTGTTACGACTTCG-3′, (*rrs* nucleotide positions 1401/1402); embB-FW, 5′- ATCGAGGTCACCTCTACCCACG-3′ and embB-RV, 5′-ATCCACAGACTGGCGTCGCTG-3′, (*embB* codon 306); rpsl-FW, 5′- ATGCCAACCATCCAGCAGCTGG-3′ and rpsl-RV, 5′- AGACCGGGTCGTTGACCAACG-3′, (*rpsl* codon 43); Ag85C-FW, 5′- ACATCAAGGTCCAGTTCCAG-3′ and Ag85C-RV, 5′-AGGTGTAGTTCTGGCCGTTGC-3′, (Ag85C codon 103). The primers used to amplify the targeted regions in *rpoB*, *katG* and *inhA* are described elsewhere [28].The following primers were used to amplify and sequence the pncA and RD105 region: pncA-FW, 5′- ATCCCAGTCTGGACACGTCG-3′ and pncA-RV, 5′- AGGAGCTGCAAACCAACTCG-3′. RD105-FW, 5′-AGTTCGATCACGGTGTCGGTG-3′ and iRD105-RV, 5′-AGCACGCCTTGATATCAGCG-3′;

iRD105-FW, 5′-AGGCAAATGTTCGACGGATACC-3′ and RD105-RV, 5′-ATCGCGAATCGTGGTGATCC-3′. Sequencing of PCR products was performed using capillary sequencing on an ABI 3730*xl* DNA analyzer by Macrogen Inc. (Amsterdam, The Netherlands).

#### Genetic characterization of strains used

Identification of drug resistance associated mutations was performed by reverse hybridisation assays [37], [38] or sequencing with primers listed in the paragraph above. Genotypic information of included strains was obtained by spoligotyping, MIRU-VNTR, IS6110-RFLP, sequencing, or whole genome sequencing [4], [32]–[34], [39], [40].

### Selection of genetic markers and design of probes

Forty-seven discriminatory markers were selected to demonstrate the potential of the MLPA assay. The markers were selected on the basis of published information and are described below. The sequence of these genetic markers was used by MRC-Holland to design probes detecting the selected markers (Table 1). The selected MLPA probes target single nucleotide polymorphisms (SNPs), wildtype sequences, RDs, small deletions or species-specific sequences. The MLPA assay was developed in stages with an initial set of 27 probes before the final set of 47 probes and one probe to control the assay was synthesised and tested.

#### Drug resistance markers

Markers associated with resistance to the first line drugs rifampicin, isoniazid, ethambutol and streptomycin, targeted by probes rpoB-176, rpoB-516, rpoB-522, rpoB-526G, rpoB-526T, rpoB-531, inhA-15, katG-315, embB-306, rpsl–43 and rpsl-88, respectively and markers identifying molecular resistance to the second line drugs fluoroquinolones, aminoglycosides and capreomycin targeted by probes, gyrA-90, gyrA-94, rrs-1401, rrs-1402, respectively were chosen on the basis of their reported *in vivo* prevalence and the importance of the drug to which they confer resistance (Table 1). All probes target the mutation conferring resistance except the probes embB-306, rpsl-43 and rrs-1401. The decision of targeting the wildtype sequence in the *emB*306 codon was based on the wide variety of resistance-conferring basepair changes reported [28]. Wildtype sequences were also targeted for the rpsl-43 and rrs-1401 loci because targeting the mutant sequence was expected to result in suboptimal specificity.

#### Genotyping markers

Markers for species identification were selected to discriminate between a broad range of lineages and sublineages of *M. tuberculosis*. We used data from a recently published and comprehensive phylogeny of MTBC strains [25], [26] and included additional genotypic markers validated in a previously published MLPA assay [28] or published elsewhere (Table 1).

These markers were selected to allow discrimination within lineages and sublineages of MTBC strains (*M. tuberculosis, M. africanum West-African 1 and M. africanum West-African 2, M. bovis, M. bovis BCG, M. canetti, M. pinnipedii, M. microti*), as well as identification of East African Indian (EAI), Central Asian (CAS), East Asian (EA) “Non-Beijing blue”, Beijing (Beijing SA+, SA−, CHIN+, CHIN−, V+, V−, K1), *M. tuberculosis* 4 (MTB4: X family, Haarlem and Latin-American/Mediterranean (LAM) [25], [26]. A lineage-specific deletion of 8bp in the *pncA* gene (nt 109–116) was targeted to identify a suspected dominant MTBC strain allegedly circulating in Bulgaria.

MLPA probes targeting RDs either hybridise to a sequence within the RD to detect an intact RD, or hybridise to the flanking regions of an RD indicating the RD is deleted (Table 1).

#### Species identification of non-tuberculous mycobacteria

Probes to detect the presence of a range of NTMs were included (*M. kansasii*, *M. xenopi*, *M. avium complex*, *M. avium* subsp. *avium* and *M. fortuitum*). These NTMs are identified by probes targeting a species-specific sequence within the 16S-23S rRNA intergenic spacer region. For identification of the non-tuberculous mycobacteria *M. xenopi*, *M. avium* subsp. *avium* and *M. fortuitum*, MLPA probes were designed containing three oligonucleotides: the left probe oligo, the right probe oligo and a sequence-specific spanning oligo in between. The introduction of a second ligation site serves as an additional discrimination point ensuring the specificity of these MLPA probes, as a standard MLPA probe (composed of two oligonucleotides) would not be sufficiently specific.

A more general probe targeting the presence of a small deletion in the 16S rRNA in MTBC [41] but absent in many NTM species was also included. An overview of the markers included and an algorithm for the identification of lineages and sublineages is shown in Table 1 and Figure 2.

## Results

From an initial list of 85 markers, 47 markers were selected for inclusion in the MLPA assay. These markers target diverse characteristics and enable discrimination within MTBC, identification of NTM species and detection of molecular resistance to anti-tuberculosis drugs. To streamline the process, probes were designed and synthesised in two rounds. We performed initial experiments to validate the first 30 MLPA probes. On the basis of the strains tested we observed that recC-1491, RD702 and RD207 were producing false-positive results or were non-functional. The recC-1491 and RD702 probes were redesigned and the RD207 probe was replaced with pckA-1119. These probes were tested along with 17 additional MLPA probes (47 probes in total) on different strains. Of the 47 MLPA probes, four probes produced false-positive results or were non-functional (RD711, pncA, rpoB-516 and rpsl-88). Thus for the strains analysed we have data from 23 or 43 MLPA probes available. MLPA products were always analysed using the 50 unique-coded beads of which 47 bead species provided information about drug resistance, species, lineage and strain identification and three bead species were reserved to monitor the quality of the assay.

Of the 47 probes, four probes were recognised as nonfunctional and excluded from the study. The 43 remaining probes are described in detail in Table 1.

**Figure 4:**
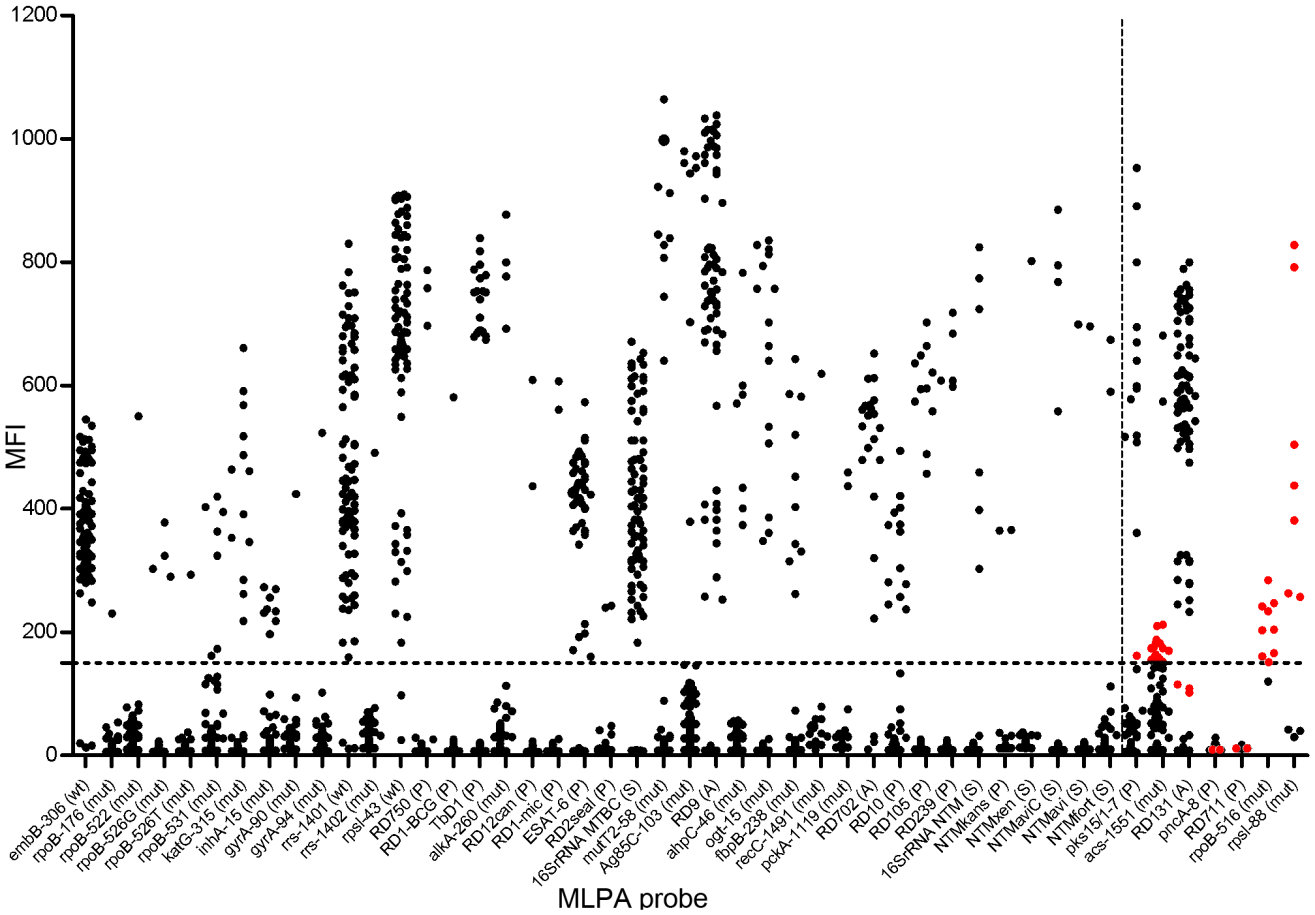
Dot plot of MLPA probe-specific MFI values of strains analysed. Median fluorescence intensity (MFI) values are indicated for each MLPA probe for every mycobacterial strain tested. The threshold used to call the presence or absence of a maker, MFI of 150, is indicated with a horizontal dashed line. Non-functional MLPA probes are indicated to the right side of the plot separated with a vertical dashed line. False positives or false negatives are highlighted in red. Brackets indicate whether a MLPA probe targets the wildtype sequence (wt), SNP (mut), the presence (P) or absence (A) of an RD, or a species-specific sequence (S).

The four nonfunctional probes target *rpoB*-D516V (rifampicin resistance), *rpsl*-K88R (streptomycin resistance), pncA (genotypic marker) and RD711 (genotypic marker). The markers rpoB-516 and rpsl-88 gave non-specific false positive results and pncA and RD711 probes were nonfunctional (Figure 4).

During validation, DNA from 89 bacterial strains was analysed by MLPA to establish the performance of 43 genotypic and drug resistance markers and the LumD and LumH control. All DNA extraction methods tested (thermolysis, CTAB method or column purification) produced interpretable results. Of the 43 functional MLPA probes, 30 were designated for species, lineage and strain identification and 13 targeted resistance associated genetic markers. Results obtained by MLPA for all 89 analysed strains were first checked for consistency and then compared to genotypic information available from sequencing, spoligotyping, line probe assays, MIRU-VNTR, IS6110-RFLP, or genetic characterization of resistance markers (Table S1, Figure 2). In total 3059 characteristics were screened of which 1915 were informative for species identification and 1144 were informative for molecular resistance to first and second line drugs (Figure 3).

Based on our initial experience with the assay we set an MFI threshold value of 150 for the presence or absence of a marker, although in principle a threshold could be independently established for each probe.

### Controls

Of the three internal controls LumQ, LumD, LumH, initially only LumD and LumH were functional and therefore used in the assay. For both controls, LumH and LumD, we obtained MFI signals higher than 300. The LumD control detected MTBC DNA and confirmed it was adequately denatured (Table S2); a positive signal with the LumH control was obtained independently of the DNA sample tested (data not shown). The LumQ control was subsequently shown to be functional when the oligo was present in the probemix at a four times higher concentration than previously used (4 times 12x10^−21^ moles) (data not shown).

The specificity of the assay was also tested on DNA from *S. aureus*. We never observed an MFI higher than the set threshold for the 16SrRNA MTBC marker, but occasionally an MFI value higher than 150 was obtained for some of the other markers.

### Internal consistency check of the MLPA markers

All 88 mycobacterial strains were checked for the consistency of MLPA markers present or absent based on the decision tree (Figure 2). In 19 strains (strain 43–46, 49, 51–52, 54–58, 60–61, 68–69, 71–72, 77) markers specific for two distinct of two lineages were found thereby causing an inconsistent call (Figure 3), this data is summarised in Table S3. The presence of the markers pks15/1–7 or acs together with a marker of another TB lineage caused an inconsistent call for these strains. These strains could therefore not be assigned to one of the lineages. For five strains (44, 54, 58, 60–61) the presence of the genotypic marker RD131 or acs alone caused an inconsistent call since these markers characterise a Beijing sublineage only together with the presence of other markers (fbpB-238 and mutT2-58).

All screened mycobacteria other than tuberculosis, *M. kansasii*, *M. xenopi* and *M. avium* subsp. *avium, M. avium* complex and *M. fortuitum*, were detected and the probes used to detect these species did not give a signal for any of the MTBC isolates tested (Figure 3). The NTM 16S rRNA marker was detected in *M. kansasii*, *M. avium* complex and *M. fortuitum* (Figure 3).

### Comparison of MLPA results to other molecular typing methods

Of 1915 characteristics screened for genotype identification, 1862 (97.2%) of them were supported by other molecular methods, 29 (1.5%) were consistent but not supported by other molecular methods and 24 (1.3%) showed discordant results when compared to other molecular methods (Figure 3).

Strain 21 was previously defined as East Asia “Non-Beijing blue” based on the presence of RD105 [25]. However, the presence of RD105 could not be confirmed by MLPA (Figure 3). This finding was confirmed by PCR-amplification of the RD105 region in strain 21. Primers could amplify the flanking regions of RD105. Based on the markers screened for strain 21 could not be assigned to any of the MTBC lineages. Spoligotyping of this strain resulted in a spoligotype pattern that also could not be designated to one of the major lineages.

Based on whole genome sequencing, strain 50 was assigned to the Beijing lineage V+/CHIN+. In addition to the markers defining the Beijing lineage V+/CHIN+ (RD105, fbpB-238 and mutT2-58), MLPA also detected a mutation in acs-1551. This strain was thus assigned to the Beijing lineage SA+ thereby causing a conflicting assignment within the Beijing lineage between MLPA and sequencing.

In the 19 strains (strain 43–46, 49, 51–52, 54–58, 60–61, 68–69, 71–72, 77) that could not be assigned to a single lineage, the presence of pks15/1–7, acs and RD131 markers in these strains detected by MLPA could not be confirmed by other molecular methods. MFI values around the set MFI threshold of 150 appeared to be the cause of these potentially false positive results (Figure 4).

The absence of the NTM 16S rRNA marker in *M. xenopi* and *M. avium* subsp. *avium* is in fact not a truly discordant result since the strains were correctly identified with the species-specific markers (strain 82, 85–86; Figure 3). The NTM 16S rRNA marker was included to discriminate a range of NTM species from the MTBC but is not able to detect all NTM species. The absence of the marker is due to variation in the sequence targeted by the MLPA probes within the different NTM species.

### Comparison of MLPA results for drug resistance to other molecular methods

A total of 13 MLPA probes (Table 1) for the detection of molecular markers associated with resistance to first and second line drugs were validated. Results were compared to data obtained by reverse hybridisation assays or post-MLPA sequencing of the targeted loci. Of the 1144 characteristics tested for drug resistance, 195 (17.0%) could be compared to previously obtained data (Figure 3). At least one positive and one negative result validated by another method was obtained for all 13 probes. Only one discordant result was identified (characteristic embB-306 in strain 10) out of the 195 characteristics tested (0.5%) for which the resistant genotype was known. This discrepancy was not due to the threshold selected but was a result of a lack of specificity of the embB-306 MLPA probe for one allele associated with resistance; in strain 10, analysis by HAIN GenoType MTBDR*sl* and post-MLPA sequencing revealed a methionine to isoleucine codon change (ATG to ATA) in the embB-306 locus whereas the MLPA produced a “wildtype” call. Previous MLPA analysis of embB306 mutant strains also demonstrated that the embB306 MLPA probe is unable to detect this ATG to ATA codon change [28].

### Reproducibility of the assay

**Figure 5:**
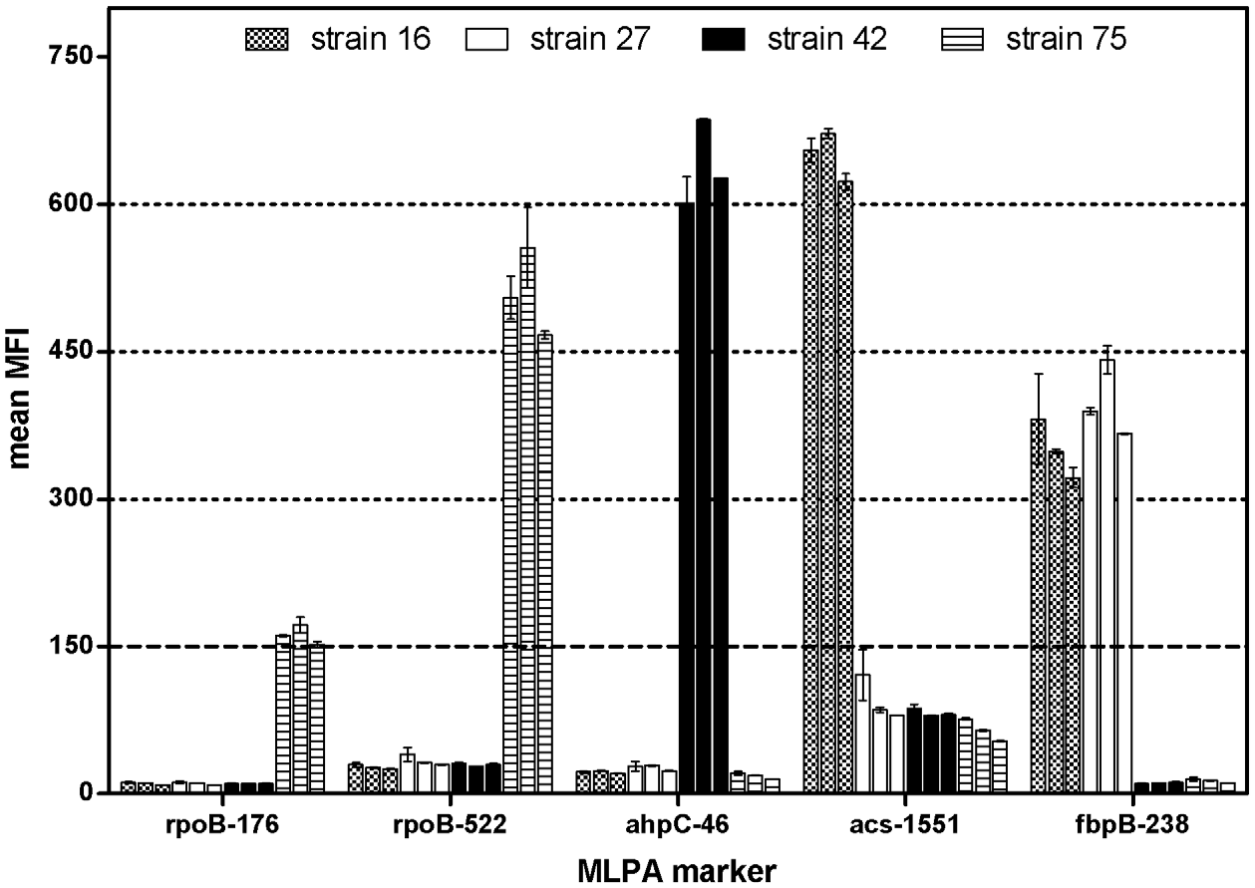
Reproducibility of the MLPA assay. DNA from *M. tuberculosis* strains 16, 27, 42 and 75 was analysed by MLPA in duplicate and in three experimental replicates. Mean Median Fluorescence Intensity (MFI) values with standard error of the mean (SEM) are shown. Each bar represents one experiment. The dashed line at MFI 150 indicates the set threshold.

For the analysis of the intra-assay and inter-assay reproducibility, four MTBC strains (strains 42, 75, 16 and 27) were analysed in duplicate by MLPA in three independent experiments (Figure 5). Intra-assay and inter-assay reproducibility were determined by calculating mean MFI values, standard variations and coefficients of variation. Two markers for drug resistance and four markers for species identification were included in the analysis (Figure 5). For all four MTBC strains the results (presence or absence of MLPA products) and the values (MFI) were reproducible within one experiment and between the three experiments. These results were concordant with previously performed MLPA analysis of these strains.

## Discussion

In this study we have validated the bead-based MLPA and thereby demonstrated the potential of MLPA to simultaneously (A) identify a range of drug resistance markers (B) discriminate between members of the *M. tuberculosis* complex (MTBC) and detect specific genetic lineages (C) detect and identify the clinically most relevant non-tuberculous mycobacterial (NTM) species *M. kansasii*, *M. xenopi*, *M. avium* complex, *M. avium* subsp. *avium*, *M. fortuitum* [42].

Several post-diagnosis methods are available, each providing detailed information on a specific trait of the infecting strain(s) [21], [37], [38], [43], [44]. However, for full characterization of MTBC isolates multiple methods are currently needed. Therefore a single multiplex method allowing simultaneous detection of a range of characteristics, including species confirmation and internal quality control would be beneficial. The rapid expansion of whole genome sequence (WGS) databases allows the rational selection of robust and informative genetic markers from a clonal organism such as *M. tuberculosis* [25], [26], [33], [34], [45], [46], which can then be directly utilised by SNP detection methods with high multiplexing abilities [31], [47].

Previously we reported the development of an *M. tuberculosis* specific molecular assay which allows the simultaneous detection of 18 discriminatory genetic markers providing information on drug resistance and bacterial lineage [28]. This method is based on Multiplex Ligation-dependent Probe Amplification (MLPA) [31] where results are read by capillary electrophoresis. Here we have expanded this MLPA assay and transferred the method to a bead-based suspension array by replacing the traditional length-coded system for the internal xTAG technology (Luminex Corp. USA), which enables readout on a MAGPIX device (Luminex Corp., USA).

The MLPA assay described here includes 12 previously described and validated markers [28] and 26 additional genotypic markers allowing the delineation of clinical isolates into the six main lineages of the MTBC [25], [26], [46] and the detection of NTMs. It further contains markers to detect resistance to streptomycin, fluoroquinolones, aminoglycosides and capreomycin, in addition to previously described isoniazid and rifampicin resistance markers [28]. These resistance markers are reported to be responsible for 53 to 62% of fluoroquinolone resistance, 71 to 97% of amikacin, kanamycin and capreomycin or amikacin, kanamycin resistance, and 22 to 64% of streptomycin resistance [48]–[57].

To validate the assay, we assessed the performance of the additional MLPA probes and transfer of the assay to the MAGPIX system by screening a well-characterised panel of 88 strains with either 23 or 43 markers, as the range of MLPA-probes was extended during the development of the assay.

Initial experiments with the bead-based MLPA showed that MFI values were sensitive to the relative concentration of the labelled, unlabelled primers and dNTPs (data not shown) but by limiting the concentration of the unlabelled primer, to one quarter of that of the labelled primer, highly reproducible MFI values could be obtained (Figure 5). Based on the MFI values obtained for 13 MLPA characteristics from analysis of 24 mycobacteria strains and two non-mycobacteria strains, we set a threshold value of 150 MFI and for all measurements with an MFI over this value we assumed an MLPA product was present. For species identification of the analysed strains we used a decision tree created on the basis of the most recent comprehensive MTBC phylogeny ([25], [26] and Figure 2). Assignment of strains to a specific MTBC lineage and determination of drug resistance was based on the presence or absence of specific markers (SNPs or RDs). Classification of the strains was done by automated interpretation of the MLPA results using a dedicated Excel sheet.

Targeting lineage-specific markers facilitates identification of specific genotypic groups, but does not generate classical “typing profiles”. MLPA results can be screened for internal consistency as each strain should only be positive for genotypic markers associated with a single lineage and negative for all other markers (Figure 2). The presence of markers from multiple lineages thus results in an inconstant profile that will easily be recognised. Such a profile would indicate either false positive or negative calls, a mixed culture, or that the selected marker is in fact not lineage-specific.

For three MLPA probes an additional central sequence-specific spanning oligo was designed, thus to obtain a positive signal three oligos must be ligated at two points. These oligos (targeting *M. xenopi*, *M. avium* subsp. *avium* and *M. fortuitum*, Table 1) targeted the rRNA interspacer region of NTM species and the introduction of a second ligation site ensured their specificity which could otherwise not have been obtained with a traditional MLPA probe.

We screened 88 strains for in total 3059 characteristics. The vast majority (3034/3059) of these characteristics were consistent with previous molecular characterizations, of which 2037/3034 were directly supported by other molecular methods and 997/3034 were consistent with but not directly supported by previous molecular characterizations. Inconsistent results, that directly conflicted or were inconsistent with previous molecular characterization, were obtained for 25/3059 of the characteristics tested (Figure 3).

The majority of the discrepant results (strains 43–46, 49–52, 54–58, 60–61, 68–69, 71–72, 77) were due to the markers pks15/1–7, acs-1551 and RD131. For these markers MFI values just above the threshold (150 MFI) were obtained in many cases (Figure 4 and Table S2), resulting in these markers being called present along with other markers specific for different lineages in some strains. Consequently these strains could not be assigned to a specific MTBC (sub)lineage by MLPA (Figure 3).

Individual adjustment of the threshold values for the markers pks15/1–7, acs-1551 and RD131 could have resolved this issue for 14 out of 20 strains with conflicting results; a single genotype, consistent with other genotyping methods, could then be assigned to these 14 strains. However, individually set thresholds would still have not resulted in assignment to a single TB (sub)lineage for strains 44, 54, 58, and 60–61, since no additional information of genotype-specific markers was present in these strains that were analysed with the initial probemix targeting only 23 markers. The markers acs-1551 or RD131 alone or in combination are not sufficient to assign a strain to the Beijing lineage. Strain 43 would have been identified by MLPA as a LAM strain but spoligotyping assigned this strain to the T family.

From this analysis we believe that automated calling of MLPA results is feasible and that future versions of the MLPA would be even more accurate if individually normalised thresholds are introduced for each marker targeted (Figure 4). The ability of a single threshold, as used here, to result in such consistent data is nonetheless encouraging and demonstrates that the reproducibility and overall signal-to-noise ratio between positive and negative markers was adequate. Signal-to-noise ratios may still be improved by further optimization of the probemix or the procedure; for example by enhancement of the positive MFI signals through exonuclease digestion of the non-labelled PCR-product [58]–[60].

As mentioned earlier, four markers were removed from the selection of 47 markers initially included in the assay (Figure 4). The 8 bp deletion in pncA was predicted to be specific for strains of the ST41 spoligotype which are highly prevalent among MDR strains in Bulgaria. The deletion was first reported in strains from Quebec, Canada [61] and the ST41 spoligotype was discovered in Turkey [62]. Retrospective comparative analysis suggested that strains belonging to the ST41 spoligotype carried the characteristic 8 bp deletion in pncA. The MLPA probe designed for detection of this deletion did not identify the two ST41 strains (strains 58 and 59, Table S1), since MFI values for the pncA probe were similar to non-ST41 strains (Table S2 and Figure 4). Subsequently sequencing confirmed this deletion was not present in these strains (results not shown). This suggests that the Quebec strain and the Bulgarian ST41 isolates have similar spoligotypes by convergence, or alternatively that the pncA deletion is specific for a branch of the ST41 strains.

Genotypic markers that allow the identification of the six main MTBC lineages were included. This broad range of markers is quite effective for the analysis of globally diverse collections of strains. However, inclusion of characteristic markers for targeting locally prevalent strains may be more informative. For instance, in East Asian countries, where Beijing genotypes are the dominant strains, increased discrimination within the Beijing lineage would be more appropriate. The MLPA assay described here included a panel of five markers that allowed further delineation of the Beijing genotype (Beijing SA+, SA−, V+, V−, CHIN+, CHIN− and K1 [33], [34]).

The ability to simultaneously detect different mycobacterial lineages and specific drug resistance would not only be useful for mapping the prevalence and spread of (drug-resistant) TB in a region, but also to track emerging and potentially more virulent genotypes. For example, a significant proportion of the MDR-TB cases in Europe are due to large clusters of a limited number of epidemic strains. In fact, 84% per cent of the clustered MDR-TB cases identified in Europe from 2003 to 2007 were caused by only seven strains, all belonging to the Beijing genotype [63]. Strains belonging to these clusters have been isolated and subjected to detailed genetic analysis which has revealed distinctive markers that identify and discriminate within the Beijing lineage [33], [34], [64]. A selection of these has been included in our assay (Table 1), among which RD131, a genomic region which is deleted in one of the largest and most widespread European MDR clusters, cluster E0054 [63]. The absence of a signal for RD131 along with the presence of two markers specific for larger subgroups of the Beijing clade (mutT2-58 and fbpB-238) allowed us to specifically identify members of the E0054 cluster (Table S2 and Figure 3; strains 1, 27 and 73).

We used Ag85C-103 to classify strains belonging to the LAM lineage [65]. From the results obtained the MLPA probe targeting this marker is functional and specific (Figure 3; strains 7, 46, 41, 52, 53). However, strain 43 was assigned to LAM by MLPA, but assigned to the T lineage by spoligotyping. Gibson et al. [65] encountered the same inconsistencies between Ag85C-103 and spoligotyping. In their study, supplemental IS6110 RFLP analysis showed that the Ag85C marker was more discriminatory for LAM strains than spoligotyping. Our results also support the finding of Abadia et al. [66] who studied two strains with a spoligotype identical to strains 60 and 61 (TUR). Their strains were initially identified as LAM7-TUR strains on the basis of a specific spoligotype signature. However, using SNP typing, Abadia et al. found their strains lacked the LAM-associated ligB SNP but contained the T sublineage TUR-T3-Osaka-associated ligC SNP. Thus, the spoligotype signature was renamed TUR as a result of “SNP-resolved spoligotyping”. The absence of the LAM-associated Ag85C-103 marker in strain 60 and 61 in our study supports the conclusion that these strains are not members of the LAM genotype.

MLPA could not confirm the classification of strain 21 which has an unusual genotype and was previously assigned to “non-Beijing blue” by MLST and LSPs (strain 21 is referred to as K100 [25], [26]). In addition, none of the standard signature patterns could be obtained for this strain by spoligotyping [25].

The MLPA is a flexible assay with regard to the inclusion of markers to be targeted; markers included in the probemix can be easily exchanged with other validated markers by replacing the target-specific sequence of individual MLPA-probes, but retaining the xTAG sequence. For some lineages multiple genotypic markers have been discovered [14], [33], [34], [46], [67], so alternative or additional SNPs or LSPs associated with the lineage targeted could be included.

Replacement of nonfunctional probes targeting specific resistance mutations is more challenging than replacement of genotypic markers, as the design of the probe is restricted to target exactly these mutations; drug resistance mutations are not equivalent to each other and the majority of drug resistance mutations have a unique prevalence in a certain geographical location. One possibility to resolve nonfunctional MLPA probes for the detection of specific drug resistance mutations is to target the wildtype locus instead. In the current assay we targeted the wildtype sequence for the indirect detection of multiple mutations in codon 306 of embB ([28] and Table 1). However, since MLPA focuses on direct detection of targeted genetic markers, targeting the wildtype sequence is most feasible if polymorphisms are confined to a single nucleotide or codon as shown for the embB-306 marker. Therefore targeting all possible mutations that are clustered in a genomic region, such as mutations in *rpoB*, is more difficult by the current MLPA procedure than for some other SNP detection methods, such as molecular beacon or line probe assays [37], [38], [44], [68], [69].

Three quality controls were included, giving information on complete denaturation (LumD), sample DNA quantity (LumQ) and efficient hybridisation of MLPA products to the beads (LumH). All three internal controls are functional and can be added directly to the DNA sample with the MLPA probemix. Further detailed analysis of the controls will reveal the performance conditions of the LumD and the detection limits of the LumQ and LumH controls.

MLPA allows lineage or strain identification, but is not a typing method and thus does not replace typing methods such as spoligotyping, MIRU-VNTR, or whole genome sequencing. Notably, high throughput methods for spoligotyping and “spoligoriftyping” have recently been developed which also utilise Luminex technology [39], [66], [70], [71] (Gomgnimbou et al, manuscript in preparation), offering the potential to perform characterization as well as comprehensive typing using the same technology.

Although profiling techniques such as MIRU-VNTR and spoligotyping in principle have a higher discrimination than SNP detection methods, like MLPA, these techniques are more prone to problems associated with homoplasy [72]. An example of this is explained above with incongruent results obtained for spoligotyping and the ligB and Ag85C SNPs and with MIRU-VNTR [73]. In addition, SNP typing is platform-independent and can therefore easily be compared between methods and laboratories and remains informative even if whole genome sequencing becomes the standard method to characterise clinical strains.

Here we describe the validation of the bead-based MLPA to characterize *Mycobacterium tuberculosis* isolates using 88 selected well-characterised strains. We have demonstrated that MLPA is a uniquely informative single test that allows the identification of MTBC members from culture as well as the detection of mutations associated with important phenotypes. Multiplex molecular methods such as MLPA produce definitive, internally consistent results and could provide real time information on an evolving tuberculosis epidemic. This type of data could previously only be obtained by detailed retrospective analysis of small collections of strains [2]. The MLPA assay appears suitable for transfer to an automated cartridge based system as it uses equipment that is robust and calling the presence and absence of markers screened was automated. The MLPA is currently being piloted in three research laboratories. The sensitivity and specificity of the MLPA assay will be evaluated in a diagnostic laboratory in a region with a high burden of MDR-TB.

## Supporting Information

Table S1 **Table shows the drug resistance profiles and species identification of all strains analysed in this study as determined by various methods.**
(XLSX)

Table S2 **Table shows marker-specific MFI values measured by the bead-based MLPA of 88 mycobacterial and one non-mycobacterial species.**
(XLSX)

Table S3 **Summary table of** **Figure 3** **.**
(XLSX)
